# Effect of low-dose hydrocortisone on gene expression profiles after severe burn injury

**DOI:** 10.1186/cc13265

**Published:** 2014-03-17

**Authors:** J Plassais, MA Cazalis, F Venet, G Monneret, A Pachot, S Tissot

**Affiliations:** 1 Joint Unit Hospices Civils de Lyon/bioMérieux, Lyon, France; 2 Hospices Civils de Lyon, Hopital Edouard Herriot, Lyon, France

## Introduction

The systemic effect of inflammatory mediators released after severe burn is a severe cardiovascular dysfunction called burn shock. Patients need vasopressor infusion to maintain adequate delivery of oxygen but it could have deleterious effects on skin perfusion and worsen the burn depth. This shock could result from the interplay of the initial hypovolemia and the release of multiple inflammatory mediators [1]. It has been shown that a low-dose of hydrocortisone could reduce the shock duration but the mechanisms involved remain unclear. We investigated the systemic genomic response after severe burn injuries and determine whether patterns of gene expression could be associated with a low dose of glucocorticoids.

## Methods

Thirty burn patients with over 30% of total body surface area were enrolled into a randomized double-blind clinical study. Fifteen patients were treated with a low dose of hydrocortisone and 15 patients were treated with placebo. Whole blood samples were collected after shock onset (S1) before any treatment, 1 day after treatment beginning (S2), and 120 hours and 168 hours after the burn injury (S3/S4). Blood samples of 13 healthy volunteers were collected. Pangenomic expression was evaluated with Affymetrix HG-U133plus 2.0 microarrays. Moderated *t *tests and *F *test were used to compare burn patients with controls and gene expression profiles between the two groups (B-H correction, *P *< 0.05).

## Results

Severe burn injury induced the deregulation of a considerable number of genes (*n *>2,200 at S1) in comparison with controls with an increased number of deregulated genes over time. Within burn patients, more than 300 genes were deregulated by hydrocortisone over time. The treatment had a rapid effect on gene expression, 339 and 627 genes were differentially expressed at S2 and S3 respectively. However, the number of these genes decreased drastically at S4 (only 24 genes significant). The genes identified at S2 were mostly related to the decrease of growth, development and quantity of leukocytes but these biological processes were not found significant at S3, indicating that the action of glucocorticoid in the response to burn injury is short lived and time dependent.

## Conclusion

This study is an informative overview of the genomic responses after burn injuries. More importantly, it is the first study providing information about mechanisms involved in glucocorticoid's reduced shock duration after burn.
